# Early Retinal Microvascular Alterations in Young Type 1 Diabetic Patients without Clinical Retinopathy

**DOI:** 10.3390/diagnostics13091648

**Published:** 2023-05-07

**Authors:** Alexandra Oltea Dan, Alin Ștefănescu-Dima, Andrei Teodor Bălășoiu, Ileana Puiu, Carmen Luminița Mocanu, Mihaela Ionescu, Andreea Cornelia Tănasie, Anca Elena Târtea, Veronica Sfredel

**Affiliations:** 1 Department of Physiology, University of Medicine and Pharmacy of Craiova, 200349 Craiova, Romaniaveronica.sfredel@umfcv.ro (V.S.); 2 Department of Ophthalmology, University of Medicine and Pharmacy of Craiova, 200349 Craiova, Romania; 3 Department of Pediatrics, University of Medicine and Pharmacy of Craiova, 200349 Craiova, Romania; ileana.puiu@umfcv.ro; 4 Department of Medical Informatics and Biostatistics, University of Medicine and Pharmacy of Craiova, 200349 Craiova, Romania; 5 Department of Neurology, University of Medicine and Pharmacy of Craiova, 200349 Craiova, Romania; anca.tartea@umfcv.ro

**Keywords:** optical coherence tomography angiography, diabetic retinopathy, type 1 diabetes

## Abstract

The purpose of this study is to identify and quantify preclinical changes with the help of optical coherence tomography angiography (OCTA) within the retinal microcirculation of young type 1 diabetes (T1D) patients without clinical signs of diabetic retinopathy (DR) and to compare these results with those obtained from healthy age-matched subjects. OCTA is currently used for monitoring diabetic retinopathy; however, there is no current consensus on which OCTA parameter alterations predict the first clinical signs of diabetic retinopathy. The main challenge that young patients with T1D face during the course of the disease is that they can rapidly progress to the development of DR, especially during adolescence. Moreover, they also present an increased risk of rapid progression toward advanced stages of DR and vision loss compared to type 2 diabetes patients, indicating the importance of early diagnosis and intervention. The limitations of the currently used screening procedures that led to the conceptualization of our study are the difficulties in performing fluorescein angiography tests for diagnosing the clinical signs of DR on young patients, namely the invasive procedure of dye injection, the risk of allergic reactions and the long duration of the examination. Moreover, given the long life expectancy of young T1D patients, it is essential to identify the preclinical changes in retinal microvasculature before reaching the first clinical signs quantifiable by FFA. The clinical study enrolled 119 subjects aged between 4 and 30 years old with a mean age of 13 years old, comprising 61 T1D patients with a mean duration of the disease of 4 years and 8 months and 58 healthy age-matched subjects for the control group. OCTA scans were performed using the RevoNX 130 OCTA device (Optopol) to evaluate the following retinal parameters: foveal avascular zone (FAZ) area, perimeter and circularity, overall foveal thickness, and superficial and deep vessel densities. Statistically significant differences between the two groups were identified for the following parameters: the FAZ area in the T1D group (0.42 ± 0.17) was larger than the control group (0.26 ± 0.080), the FAZ circularity (0.41 ± 0.11) was decreased compared to the control group (0.61 ± 0.08) and the FAZ perimeter was larger (3.63 ± 0.97) compared to the control group (2.30 ± 0.50). The overall foveal thickness was decreased in the T1D group (222.98 ± 17.33) compared to the control group (230.64 ± 20.82). The total vessel density of the superficial capillary plexus (SCP) on an investigated area of 6 X 6 mm centered around the fovea was decreased in the T1D group (37.4164 ± 2.14) compared to the control group (38.0241 ± 2.44). Our data suggest that specific imaging biomarkers such as FAZ perimeter, area and circularity, decreased overall foveal thickness and decreased vessel density in the SCP precede the clinical diagnosis of DR in young T1D patients and represent useful parameters in quantifying capillary nonperfusion in T1D patients without clinical signs of DR.

## 1. Introduction

Diabetes Mellitus represents a major public health priority, currently affecting more than 425 million people worldwide [1], with approximately 8.4 million people being affected by type 1 diabetes [2].

Type 1 diabetes (T1D) is a chronic immune-mediated disorder characterized by the destruction of pancreatic β cells leading to absolute insulin deficiency and hyperglycemia [3]. It is the third most frequent chronic disease among children [4], with an annual increase in incidence and prevalence of about 2–3% per year [2]. The highest increase in incidence affects children younger than 15 years, especially those younger than 5 years old [5,6].

Despite the known genetic predisposing factors, the high increase in incidence is attributed to both environmental and behavioral factors. The known behavioral factors are attributed to increased urbanization and include poor diet and lack of physical activity, lack of D3 vitamins and nitrosamines in processed food. Endocrine-disrupting chemicals found in processed food can accelerate T1D through immunomodulation and gut microbiota. [7] The most frequent environmental factors associated with type 1 diabetes include viruses associated with islet inflammation, decreased gut microbiome diversity and decreased vitamin D levels [7].

Diabetic retinopathy (DR) is the most common microvascular complication of T1D and one of the leading causes of irreversible blindness even in developed countries [7,8]. The prevalence of DR among diabetic patients is 34.6% worldwide, meaning approximately 93 million people are diagnosed with diabetic retinopathy, 21 million of them with diabetic macular edema and 17 million with proliferative diabetic retinopathy [9].

While the main risk factors for the development of DR are the duration of the disease and poor glycemic control, the other risk factors that need to be taken into consideration for T1D patients include puberty and pregnancy, as well as genetic heritability and glycemic variability [10].

T1D adolescents can present a high risk of rapidly progressing towards sight-threatening retinopathy compared to adults with T1D [11] through clinically significant macular edema and proliferative retinopathy [12].

T1D is currently known to be more aggressive than type 2 diabetes (T2D), with a higher prevalence of microvascular complications. It is therefore essential to detect and quantify the microvascular damage preceding the clinical signs of DR for patients with T1D, in order to delay or even prevent advanced retinopathy [13] for children and young adults affected by T1D.

The first visible clinical signs of DR are characterized by the presence of microaneurysms, large vessel dilation and intraretinal hemorrhages, all easily identifiable on fundus examination. The deep retinal and choroidal alterations that precede the onset of these lesions cannot be visualized on fundus examination.

Multiple imaging modalities such as dilated fundus examination, color fundus photography, fundus fluorescein angiography (FFA) and optical coherence tomography (OCT) are currently used for diagnosing and staging DR. The most accurate method for diagnosing and staging DR over the years was FFA, offering detailed information about retinal vascular integrity. However, FFA is a dye-based invasive test, presenting several risks and contraindications, especially for young T1D patients.

In recent years, new and noninvasive imaging techniques such as optical coherence tomography angiography (OCTA) are allowing better visibility of the deep retinal and choroidal structure and vasculature using a contrast-free technique. Compared to FFA, OCTA allows the visualization and objective quantification of the separate layers of retinal and choroidal blood flow including precise areas of capillary non-perfusion, the presence of collateral vessels and abnormalities of the foveal avascular zone (FAZ) without the need for dye injections [14]. OCTA is unique in separately analyzing each of the retinal capillary layers, the superficial capillary plexus (SCP), the intermediate capillary plexus and the deep capillary plexus (DCP), allowing a better understanding of the pathophysiological changes in DR. The OCTA images are three-dimensional (3D), offering detailed information about early microcirculation disorders [15].

As artificial intelligence (AI) technologies develop, ophthalmology has benefited from these advancements, due to the availability of multiple image-based investigations. At the moment, AI in ophthalmology is mostly used to improve disease diagnoses such as DR, glaucoma, age-related macular degeneration (AMD) and other anterior segment diseases [16].

Along with DR, other major chronic diseases such as myocardial infarction and stroke are benefiting from significant advances in AI analysis, aiming to detect the key clinical characteristics of patients, their major health risks and the course of the disease after treatment. Machine learning (ML) algorithms that analyze clinical features such as gender, age, body mass index, hypertension, history of heart disease and smoking status have proven efficient in assisting the diagnosis process and improving the treatment plan. Moreover, the ML ability to process massive amounts of data by recognizing patterns and finding prediction models can lead to better decision making and decreased practice variation and can even predict the risk of myocardial infarction or stroke [17]. Multiple artificial intelligence-based technologies are also being developed to detect and grade DR at the initial level. Therefore, vision-threatening complications can be avoided through proper treatment protocols. The latest deep learning (DL) technologies in optics construct automated DR detection tools that can improve universal care for DR screening [18].

DL techniques based on interpreting OCT scans have achieved reliable results in ophthalmology in recent years; however, AI is not widely used in clinical practice yet.

Although numerous clinical studies have focused on retinal blood flow alterations in adult type 2 diabetic patients, only a few of them have researched the retinal changes in young patients with T1D.

This study aims to identify and quantify the preclinical changes observed with the help of OCTA scans within the retinal microcirculation and structure of young T1D diabetic patients without clinical signs of diabetic retinopathy and to compare these results with those obtained from healthy age-matched subjects.

For our study, we elaborated the inclusion criteria for both study groups. Next, we recruited and investigated the patients using the OCTA, over a period of 12 months. Subsequently, we reviewed the scans and considered only the high-quality ones for analysis. Then, we performed the statistical analysis of the retinal parameters.

## 2. Materials and Methods

### 2.1. Patients

This retrospective, exploratory study included 119 subjects and was carried out at the Departments of Pediatrics and Diabetes of the Clinical Emergency Hospital of Craiova and ATB Ophthalmology Clinic, Craiova, Romania. The clinical study enrolled 61 patients aged between 4 years old and 30 years old diagnosed with T1D, monitored in the Departments of Pediatrics and Diabetes of The Clinical Emergency Hospital Craiova, who met the inclusion criteria and agreed to participate, and 58 healthy subjects for the control group. The duration of the study was 12 months.

The study protocol was approved by the Ethics Committee of the University of Medicine and Pharmacy Craiova (Project Identification Code 8612/07/06/2021) and was carried out in accordance with the rules of the Declaration of Helsinki, revised in 2013. Written informed consent was obtained from all the patients who met the inclusion criteria and the legal guardians of patients younger than 18 years old, before they participated in the study.

The control group members were recruited from subjects who visited the ophthalmology clinic for a routine ophthalmic examination. They were from the same age group and had no ocular or systemic pathology, except for spherical or cylindrical refractive errors no higher than 1.5 diopters.

The inclusion criteria for the study T1D group were as follows: patients diagnosed with T1D according to the criteria of the International Society for Pediatric and Adolescent Diabetes (ISPAD), with no clinical signs of DR and a duration between the onset of the disease and the time of the investigation of at least one year, without other ophthalmic or systemic disorders, except for spherical or cylindrical refractive errors no higher than 1.5 diopters. The inclusion criteria for the control subjects were as follows: written informed consent, no systemic disorders and no ophthalmic disorders except for spherical or cylindrical refractive errors ≤ 1.5 diopters.

The exclusion criteria for both study groups were as follows: a history of retinopathy of prematurity, hereditary retinal dystrophies, vitreoretinal diseases, other fundus abnormalities including DR, optic nerve disorders, uveitis, ocular trauma, previous ocular surgery, ocular media opacities including cataracts or any other ocular or systemic disorders.

**Figure 1:**
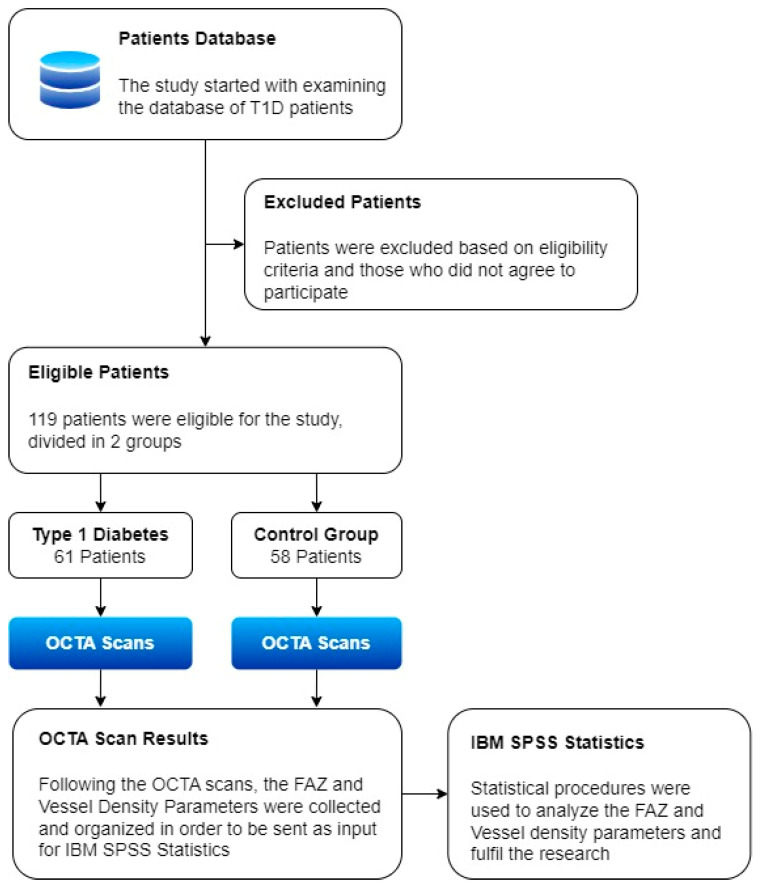
Study workflow.

The study workflow is defined in Figure 1.

### 2.2. Ophthalmic Examination

Patients from both groups underwent a comprehensive ophthalmic examination using the following tests: best-corrected visual acuity (BCVA) (LogMAR), intraocular pressure measurements using the IC 100 ICare tonometer, ICare, Vantaa, Finland, slit-lamp biomicroscopy examination and dilated fundus examination using tropicamide 1% eye drops. Refractive measurements were carried out with the help of a Nidek Ark-1 refractor–keratometer device.

The RevoNX 130 OCTA device (Optopol, Zawiercie, Poland) was used to evaluate retinal microcirculation. The scans were performed on dilated eyes using 1% tropicamide eye drops. Multiple scans were captured for both eyes, and the best-quality image was analyzed. The OCTA system used for the study relies on an algorithm of split-spectrum amplitude-decorrelation angiography. The scans captured an area of a 6 mm × 6 mm centered on the fovea. The OCTA software automatically segments the analyzed area into the superficial capillary plexus (SCP), deep capillary plexus (DCP), outer retinal layer and choriocapillaris layer.

The OCTA software automatically analyzed the following parameters: foveal avascular zone (FAZ) area, perimeter and circularity (the ratio of the perimeter of FAZ and the perimeter of a circle with equal area), overall foveal thickness, and superficial and deep vessel densities (the vessel density is defined as the total area of perfused vasculature per unit area in a region of measurement). Two experienced investigators reviewed the OCTA images taken for both groups to ensure the inclusion of high-quality scans for further analysis. Poor-quality scans with blink artifacts, motion artifacts and low signal strength were excluded from the analysis.

### 2.3. Statistical Analysis

The data collected from the reports obtained following the RevoNX 130 OCTA investigation were initially processed using Microsoft Excel 2019 (San Francisco, CA, USA), and arranged into subgroups. Continuous parameters were defined as mean ± standard deviation (SD), as well as minimum and maximum values. Categorical variables were indicated as numbers and percentages. Statistical tests were applied using Statistical Package for Social Sciences (SPSS), version 20 for Windows (IBM Corp., Armonk, NY, USA). Normality testing was based on graphical methods (including Normal Q-Q Plots and the use of histograms), and on numerical methods, namely the Shapiro–Wilk test for normality, recommended for smaller sample sizes. Group comparisons were performed as follows: an independent sample *t*-test for normally distributed data, and a Mann–Whitney U test otherwise. The value *p* < 0.05 was defined as statistically significant.

## 3. Results

The current study included 119 subjects aged between 4 and 30 years old, with an overall mean age of 13.13 ± 5.025 years old. They were distributed into 61 patients with T1D and 58 subjects belonging to the control group. The two groups had similar age and gender distributions (*p* > 0.05), as indicated in Table 1.

**Figure 2:**
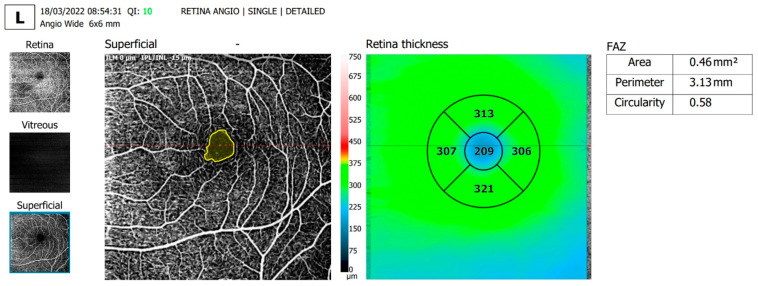
Representative images of a T1D patient with a duration of the disease of 10 years, showing enlarged FAZ area and perimeter, decreased FAZ circularity and decreased foveal thickness.

**Figure 3:**
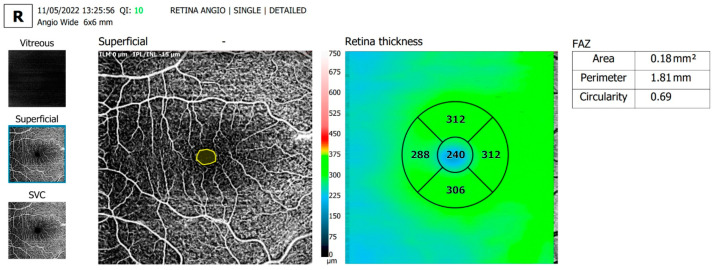
Representative images of a female subject of similar age from the control group, showing normal FAZ parameters and foveal thickness.

**Figure 4:**
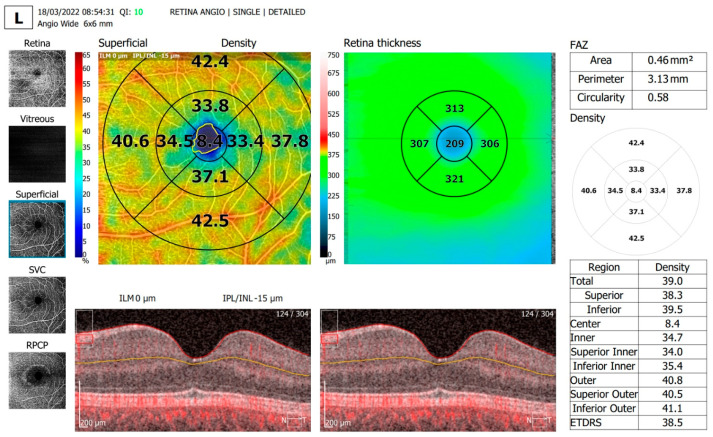
Vessel density assessment parameters in the superficial capillary plexus, in an area of 6 × 6 mm centered around the fovea in the same T1D patient.

**Figure 5:**
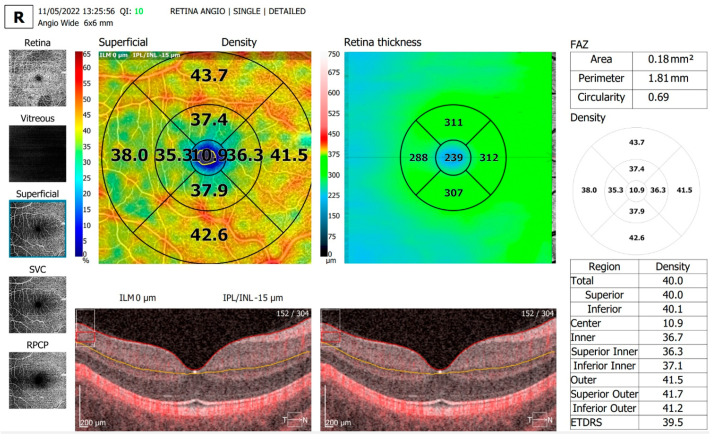
Vessel density assessment parameters in the superficial capillary plexus, in an area of 6 × 6 mm centered around the fovea in the same control subject.

The OCTA parameters evaluated and shown in Table 2 were FAZ area, FAZ perimeter and FAZ circularity, overall foveal thickness (Figure 2 and Figure 3) and vessel density in the SCP on an area of 6 × 6 mm centered around the fovea (Figure 4 and Figure 5).

Of the 18 analyzed parameters, FAZ circularity, superficial region inner, superficial region inferior inner and deep region center were normally distributed, as assessed by the Shapiro–Wilk test (*p* > 0.05). For these parameters, an independent samples *t*-test was conducted to determine whether there were differences in the associated values between the T1D group and the control group. There were no outliers in these data, as assessed by an inspection of boxplots. There was homogeneity of variances, as assessed by Levene’s test for equality of variances (*p* > 0.05). For the other 14 parameters, a Mann–Whitney U test was run to determine whether there were differences between the T1D group and the control group. Distributions of these parameters for patients with and without T1D were similar, as assessed by visual inspection.

Following these group comparisons, statistically significant differences between the two groups were identified for the following parameters: FAZ area, FAZ circularity, FAZ perimeter, overall foveal thickness, and vessel density in the SCP in the superior and inferior region, as indicated in Table 2. Data are represented as mean ± standard deviation, and min–max interval.

The FAZ area in the T1D group (0.42 ± 0.17) was larger than the control group (0.26 ± 0.08), the circularity of the FAZ in the T1D (0.41 ± 0.11) was decreased compared to the control group (0.61 ± 0.08) and the FAZ perimeter was larger in the T1D group (3.63 ± 0.97) compared to the control group (2.30 ± 0.50). The overall foveal thickness was decreased in the T1D group (222.98 ± 17.33) compared to the control group (230.64 ± 20.82). The total vessel density of the superficial capillary plexus (SCP) on an investigated area of 6 X 6 mm centered around the fovea was decreased in the T1D group (37.4164 ± 2.14) compared to the control group (38.0241 ± 2.44). The specific regions in the SCP with statistically significant differences were the superior and inferior regions compared to the control group. There were no statistically significant differences between the two groups for the DCP vessel density parameters.

### T1D Group Analysis

For the diabetic group, which comprised 61 patients, the duration of T1D varied from 1 year to 20 years, with a mean duration of 56 months (4 years and 8 months), as indicated in Table 3. A Mann–Whitney U test was conducted to determine whether there were differences in T1D duration between females and males. Distributions of the duration for these groups were not similar, as assessed by visual inspection. T1D duration for females was higher (mean rank = 31.46) than for males (mean rank = 30.70); however, it was not statistically significantly different, U = 433, z = −0.163, *p* = 0.871. The patient’s age at the onset of T1D varied from 1 year and 3 months old up to 15 years old, with an average of 91 months (7 years and 7 months old). There were no statistically significant differences regarding the age at onset between genders (U = 541, z = 1.434, *p* = 0.152). The average weight of the T1D patients at the time of the OCTA investigations was 45.97 ± 20.39 kg and the average height was 149.1 ± 20.42 cm, with an average BMI value of 19.70 ± 4.75 kg/m^2^. The average HbA1C values at the onset of the disease were 12.16 ± 2.18 (%) and the mean values over the duration of the disease were 7.53 ± 1.42%, as shown in Table 3.

We aimed to investigate whether the duration of the T1D could be correlated to the retinal microvascular changes of our studied patients. We divided the patients into two subgroups: 42 patients (representing 68.85%) with a duration of less than 5 years (60 months), and 19 patients (representing 31.15%) with a duration of more than or equal to 5 years. There were no statistically significant differences between the two subgroups regarding the FAZ and SCP parameters, as shown in Table 4.

## 4. Discussion

The main concern in diagnosing diabetes at a young age is the development of complications at an earlier stage of life [19]. DR is the most serious eye complication of diabetes, with a complex underlying pathogenesis that is not yet fully clarified.

For T1D patients, the presence of DR leads to a higher risk for vision loss as the duration of diabetes increases. Early OCTA retinal screening is preferred to FFA for young T1D patients due to its noninvasive character and detailed imaging properties, thereby reducing the risk of vision loss by identifying the preclinical retinal alterations [20].

The macroscopic retinal changes of DR are considered the end result of many years of cumulative microscopic capillary pathology undetectable on ophthalmoscopy [21]. OCTA can capture these early capillary changes, being a valuable diagnostic tool for T1D patients. The superiority of OCTA over FFA consists in its ability to detect capillary non-perfusion, as FFA does not show the details of retinal capillaries while OCTA detects the capillaries themselves and is not sensitive to the level of choroidal fluorescence or to any leakage [22].

In the current study, we investigated and compared the following retinal OCTA parameters in young patients diagnosed with T1D, without any clinical sign of diabetic retinopathy and an age-matched control group: FAZ area, FAZ perimeter, FAZ circularity, foveal thickness, and SCP and DCP vessel densities. The FAZ parameters and vessel densities are related to the retinal functionality, while foveal thickness offers information about the retinal structure.

The normal FAZ in healthy subjects has a circular or slightly elliptical shape which becomes increasingly irregular in patients with DR [23]. The FAZ circularity also impacts the FAZ perimeter and FAZ area [24]. Our study revealed statistically significant differences between the two groups for the following: FAZ area, FAZ circularity and FAZ perimeter. From this perspective, our results are in line with previous clinical studies. Merve Inanc and associates [13] also found significant statistical differences in the mean values for the FAZ perimeter and circularity index in the T1D group compared to the control group. Similarly, their results showed no significant correlations between the T1D duration and the investigated OCTA parameters. Takase and associates demonstrated a statistically significant enlargement of the FAZ area in diabetic patients compared with nondiabetic ones, regardless of the presence of DR [25]. On the other hand, Golebiewska and associates found normal vessel densities in superficial and deep plexuses, as well as normal FAZ parameters in young patients with T1D compared to healthy subjects [26].

Our study confirms previous findings that patients with T1D without DR have a larger FAZ area, perimeter and decreased circularity compared to the control group, and adds knowledge by demonstrating statistically significant differences in overall foveal thickness and vascular densities of the SCP between the two study groups. It also demonstrates statistically significant differences in vascular density within certain regions of the SCP, the superior and the inferior region. The analytic software of the OCTA allowed vessel density evaluation in the SCP and the DCP; however, there were no significant differences regarding the DCP between the two study groups. When dividing the T1D group into two subgroups according to the duration of the disease, 42 patients with a T1D duration of less than 5 years and 19 patients with a T1D duration of more than 5 years, we did not find significant statistical differences in the FAZ and SCP parameters.

The strengths of our study consist of the noninvasive evaluation of retinal capillary perfusion of the posterior pole and mid-periphery in young T1D patients without clinical signs of DR, the precise automatic segmentation measurements and the automatic data analysis. Moreover, the young age group of the patients and strict exclusion criteria suggest that the microvascular retinal alterations detected with OCTA are not related to other systemic vascular pathologies that are frequently encountered in older, type 2 diabetic patients, offering relevant results for future screening criteria for DR. In addition, the OCTA scans used in the study focus on a larger area around the fovea of 6 mm × 6 mm.

The limitations of the current study consist of the relatively small number of participants and the difficulty in obtaining high-quality images from young children. Moreover, the duration of T1D in our studied patients is a short one, with a mean value of 4 years and 8 months. There is a disproportion between the two T1D duration subgroups: the subgroup with a duration of up to 5 years included 42 patients, while the group with a duration of more than 5 years included only 19 patients. Another limitation of our current study is that the patients were not monitored over a longer period of time with OCTA scans taken yearly to assess the progression of the retinal changes. From an environmental point of view, another limitation of the study is related to the medical imaging process being a source of CO2 equivalent emission. Although it may seem that individual imaging tests are insignificant regarding the environmental impact, on a global scale, billions of imaging tests each year lead to a significant environmental footprint with major implications regarding global health risks and societal costs [27]. Therefore, we aim to avoid the overuse of medical imaging for our patients, for both diagnosis and research purposes.

## 5. Conclusions

Our data suggest that alterations in the FAZ perimeter, area and circularity, as well as decreased overall foveal thickness and decreased vessel density in the SCP, precede the clinical diagnosis of DR in young T1D patients. The outlined OCTA parameters demonstrate alterations in retinal microcirculation, as indicated by FAZ parameters and vessel density, and alterations in retinal structure, as indicated by the overall foveal thickness. These outcomes can be regarded as specific imaging biomarkers in future screening programs of T1D patients and detect early DR changes with the help of deep learning algorithms and artificial intelligence. The vessel density of the SCP in the parafoveal region is a useful parameter in quantifying capillary nonperfusion in T1D patients with no clinical signs of DR.

We aim to extend the current study by adding new members of the T1D group with a duration of more than 5 years. Moreover, we intend to take yearly OCTA scans over a period of 5 years for the diabetic patients and monitor the progression of the investigated retinal parameters. We also plan to further investigate any correlations between the altered retinal parameters and other metabolic parameters such as glycated hemoglobin and body mass index.

OCTA represents a reliable, noninvasive, dyeless method to detect early retinal blood flow alterations, especially in young T1D patients without clinical signs of DR, by rapidly assessing retinal microvascular alterations. They may also reflect similar microvascular changes occurring elsewhere in the body, such as renal glomerulus. Therefore, early detection of retinal microcirculation and structure alterations with the help of OCTA may lead to a different approach in the overall clinical management of T1D patients.

The results of our study are intended to help reach a consensus regarding the first OCTA parameters, which are modified in the retinal microcirculation of T1D patients without clinical signs of diabetic retinopathy. Young diabetic patients could be screened with the help of OCTA, and by early identification of retinal alterations they could improve metabolic control and delay the progression towards advanced stages of diabetic retinopathy.

## Figures and Tables

**Table 1:** Demographic characteristics of study participants.

Group	Participants			Age (Mean ± SD) (Years)		
	F	M	Overall	F	M	Overall
Control group	30 (55.56%)	28 (43.08%)	58	14.00 ± 5.589	13.96 ± 4.842	13.98 ± 5.196
T1D group	24 (44.44%)	37 (56.92%)	61	12.00 ± 5.898	12.54 ± 3.927	12.33 ± 4.760
TOTAL	54 (100%)	65 (100%)	119	13.11 ± 5.762	13.15 ± 4.367	13.13 ± 5.025

**Table 2:** FAZ parameters, vessel density tool parameters in different sections and foveal thickness assessment for the two groups.

Parameter	T1D Group Mean ± SD (min–max)	Control Group Mean ± SD (min–max)	*p*
FAZ Area	0.4238 ± 0.1755 (0.14–1.27)	0.2695 ± 0.08495 (0.09–0.60)	<0.0005 *
FAZ Circularity **	0.4148 ± 0.1105 (0.22–0.68)	0.6193 ± 0.0849 (0.34–0.79)	<0.0005 **
FAZ Perimeter	3.6334 ± 0.9762 (2.07–7.24)	2.3026 ± 0.5015 (1.32–3.86)	<0.0005 *
VD Superficial Region Total	37.4164 ± 2.1424 (30.70–41.30)	38.0241 ± 2.4478 (28.80–41.30)	0.037 *
VD Superficial Region Superior	37.5885 ± 2.2630 (31.30–41.70)	38.0707 ± 2.9022 (27.60–42.50)	0.043 *
VD Superficial Region Inferior	37.3295 ± 2.5328 (28.30–40.80)	37.8793 ± 3.0998 (25.80–41.00)	0.024 *
VD Superficial Region Center	13.5475 ± 5.1868 (4.40–28.80)	13.0828 ± 6.5091 (3.60–35.20)	0.413 *
VD Superficial Region Inner **	36.2656 ± 3.2766 (29.60–42.80)	35.4862 ± 3.6417 (26.10–42.50)	0.222 **
VD Superficial Region Superior Inner	36.7754 ± 3.4899 (27.80–43.10)	35.8155 ± 4.2219 (21.00–42.90)	0.239 *
VD Superficial Region Inferior Inner **	35.7426 ± 3.4031 (29.60–42.60)	35.1621 ± 3.8549 (26.20–42.50)	0.385 **
VD Deep Region Total	41.5738 ± 1.5151 (36.90–43.70)	41.2690 ± 2.3644 (28.80–43.80)	0.532 *
VD Deep Region Superior	41.6672 ± 1.8051 (35.90–44.10)	41.1466 ± 2.7237 (27.40–43.90)	0.312 *
VD Deep Region Inferior	41.4049 ± 1.8256 (33.20–43.50)	41.3276 ± 2.6060 (30.00–44.20)	0.520 *
VD Deep Region Center **	29.9246 ± 4.2637 (21.30–37.70)	29.2534 ± 4.9685 (13.20–39.50)	0.430 **
VD Deep Region Inner	43.1246 ± 1.1856 (38.30–45.20)	42.7293 ± 1.8152 (35.70–45.10)	0.413 *
VD Deep Region Superior Inner	43.3098 ± 1.1874 (38.90–45.50)	42.7483 ± 2.1036 (32.40–44.80)	0.203 *
VD Deep Region Inferior Inner	42.9328 ± 1.4653 (35.80–44.90)	42.6966 ± 2.1772 (33.20–45.40)	0.968 *
Overall Foveal Thickness	230.64 ± 20.8200 (190.00–298.00)	222.98 ± 17.3360 (190.00–269.00)	0.046 *

* Mann–Whitney U test, ** *t*-test.

**Table 3:** Investigative traits of T1D study participants.

Investigative Traits	T1D Group
T1D duration (mean ± SD) (months)	56.05 ± 53.450
Age at onset of T1D (years old)	7.34 ± 3.80
Weight (kg)	45.97 ± 20.39
Height (cm)	149.1 ± 20.42
BMI (kg/m^2^)	19.70 ± 4.75
HbA1C at onset (%)	12.16 ± 2.18
HbA1C mean value (%)	7.53 ± 1.42

**Table 4:** FAZ and vessel density assessment tool parameters and T1D duration.

Parameter	T1D Duration (Mean ± SD)		
	<5 Years	≥5 Years	*p*
Area	0.4281 ± 0.1937	0.4142 ± 0.1306	0.938 *
Circularity **	0.4188 ± 0.1176	0.4058 ± 0.0951	0.674 **
Perimeter	3.6440 ± 1.0771	3.6100 ± 0.7301	0.651 *
Superficial Region Total	37.1524 ± 2.2555	38.0000 ± 1.7857	0.168 *
Superficial Region Superior	37.3333 ± 2.4663	38.1526 ± 1.6517	0.246 *
Superficial Region Inferior	37.0810 ± 2.6469	37.8789 ± 2.2275	0.265 *
Superficial Region Center	13.2714 ± 5.5036	14.1579 ± 4.4847	0.323 *
Superficial Region Inner **	35.7524 ± 3.4983	37.4000 ± 2.4367	0.069 **
Superficial Region Superior Inner	36.3357 ± 3.8589	37.7474 ± 2.2867	0.175 *
Superficial Region Inferior Inner **	35.1381 ± 3.5160	37.0789 ± 2.7760	0.058 **
Deep Region Total	41.3643 ± 1.6629	42.0368 ± 1.0122	0.103 *
Deep Region Superior	41.5024 ± 1.9724	42.0316 ± 1.3400	0.354 *
Deep Region Inferior	41.1405 ± 1.9650	41.9895 ± 1.3378	0.063 *
Deep Region Center **	29.3262 ± 4.1300	31.2474 ± 4.3660	0.104 *
Deep Region Inner	42.9833 ± 1.3079	43.4368 ± 0.7994	0.207 *
Deep Region Superior Inner	43.1762 ± 1.3207	43.6053 ± 0.7699	0.230 *
Deep Region Inferior Inner	42.7762 ± 1.6292	43.2789 ± 0.9629	0.221 *

* Mann–Whitney U test, ** *t*-test.

## Data Availability

The authors declare that the data for this research are available from the correspondence authors upon reasonable request.
